# Ascaris Megalocephalis in the Hog

**Published:** 1896-06

**Authors:** A. Youngberg

**Affiliations:** Lake Park, Minn.


### ASCARIS MEGALOCEPHALIS IN THE HOG.

By A. YOUNGBERG, D.V.S.,
LAKE PARK, MINN.

On February 28, 1896, I was called to examine a drove of swine, numbering seventy-five, at Glyndon, Minnesota. Prior visits to this farm always found the swine in good condition, but on this occasion they were in extremely poor condition. Inquiry revealed the fact that they had been in ill-health two months. The attendant informed me that a mixture of salt, sulphur, ashes, and charcoal had been administered. Fall pigs seemed to suffer most.

*Symptoms.* Hair standing upright; very much emaciated; some were coughing; others paralyzed and unable to walk. One lot, numbering seven of the poorest in flesh, were separated from the others and had received daily a half-tablespoonful of carbolic acid and a tablespoonful of turpentine in linseed oil, but without any noticeable results.

*Autopsy.* I requested the destruction of one of this lot for autopsy, and examination revealed the presence of myriads of ascarides, both in the stomach and large intestine. Another was destroyed, and revealed the existence of large numbers of these parasites. The mucous membrane of the stomach and large intestine was highly congested, and the peritoneum presented a similar appearance.

*Treatment.* Ordered complete abstinence from food for thirty-six hours, after which powdered areca-nut was administered in milk, and followed three hours later with a large dose of chloroform and castor oil. This treatment was repeated forty-eight hours later. A letter received from the farm later reported the treatment as effective in bringing from the animals large numbers of the worms, some alive, others dead.

---

Dr. Charles F. Dawson, of Washington, D. C., recently discovered an outbreak of swine-tuberculosis in Maryland.

The weight of opinion seems to favor the prompt removal of the calf from the mother as soon as born, and feeding the calf artificially. In young stock it is claimed that nothing tends to restrict the capacity of the udder and lessen the amount of milk produced more than the custom of allowing the calves to suckle the mother.

Powdered horse-chestnut has proved very beneficial in another case of pulmonary emphysema of a year's standing during the past month at the Philadelphia Veterinary Sanitarium. Half-ounce doses were given twice daily in mixed feed, with an almost complete abatement of the distressed breathing so marked at the onset.

